# Adolescents in the Dunkelfeld: A Study of Help Seeking Minors Who Use Child Sexual Abuse Materials

**DOI:** 10.1177/10790632251351254

**Published:** 2025-06-16

**Authors:** Viola Westfal

**Affiliations:** 1 Charité – Universitätsmedizin Berlin, Corporate Member of Freie Universität Berlin and Humboldt-Universität zu Berlin, Institute of Sexology and Sexual Medicine, Berlin, Germany

**Keywords:** child sexual abuse material, adolescents, causer-related prevention, help-seeking

## Abstract

This study examines the characteristics of help-seeking minors (*n* = 117) who have consumed *child sexual abuse material* (CSAM) and who voluntarily sought support. 97% of the respondents were in *Dunkelfeld* at the time of data collection. About half of the respondents reported a pedohebephile sexual preference. The study found two distinct groups based on cluster analysis, primarily distinguished by their mental well-being. The results indicate that 60.7% of the participants had used CSAM in the last two weeks, with the most common content being medium severity on the COPINE scale (levels 6–9). The study suggests that minors are already aware of the problem and motivated to confront their problematic behavior. However, limitations such as potential bias due to self-reporting, self-selection and lack of control mechanisms must be considered. The findings underscore the necessity for the implementation of targeted prevention and intervention measures for affected groups, as well as for further research to validate and expand upon these findings.

## Introduction

The seemingly unstoppable rise in the availability and circulation of *child sexual abuse material* (CSAM) has brought the issue to the forefront of political, legal, and scientific discourse. Research studies frequently introduce the topic by citing drastic increases in criminal statistics and reports of CSAM-related incidents. However, figures featuring individuals who are considered perpetrators are less common. This study adopts a person-centered approach to shed light on an under-researched group of individuals who may occupy a dual role as both users and victims, namely, young people who engage with CSAM or so-called “child pornography”. According to the Terminology Guidelines for the Protection of Children from Sexual Exploitation and Sexual Abuse (2016), the terms “child sexual abuse material” (CSAM) or “child sexual exploitation material” (CSEM) are preferred over the term “child pornography” to emphasize the abusive nature of these materials and to distinguish them from the euphemistic nature of the term “pornography”. Nevertheless, the term “child pornography” remains prevalent in legal contexts. The terms CSAM and CSEM are not entirely congruent: CSAM can largely be understood as a synonym for “child pornography”, as the term describes material that documents the sexual abuse of children. CSEM is a broader term and goes beyond CSAM, as material that does not depict sexual conduct involving children can also be exploitative. In this context, the term “CSAM” is used because it refers unambiguously to illegal material and is commonly used in recent scientific studies.

### Prevalence and Trends in Exposure to Child Sexual Abuse Material

Various studies have shown that up to 13.5% of the general adult population, primarily males, have used CSAM or would do so if there were no negative consequences (Bártová et al., 2021; Dombert et al., 2016; Napier, 2022; Wurtele et al., 2014). These findings are based on large-scale general population samples from different countries: Bártová et al. (2021) conducted a nationally representative survey of the Czech population (*n* = 10,044, including 5023 men and 5021 women aged 18+), focusing on the prevalence of paraphilic interests. The study found that 3.1% of men and 1.6% of women consumed CSAM involving prepubescent children in the past six months; for CSAM depicting peripubescent children, the rates were 9.7% for men and 1.7% for women. Dombert et al. (2016) assessed self-reported sexual interest in children among a sample of 8718 adult German men. The study’s objective was to estimate the prevalence of sexual fantasies and behaviors involving prepubescent children. The results indicated that 1.7% of respondents reported having consumed CSAM. Napier (2022) conducted an anonymous online survey of *n* = 5512 English-speaking adults of all genders to explore the characteristics of CSAM users. Of those surveyed, 13.5% self-reported having viewed CSAM. A study by Wurtele et al. (2014) involving *n* = 435 U.S. adults (18+, males and females) examined the extent to which non-incarcerated and non-clinical adults reported sexual interest in children. The study found that 9% of male participants and 3% of female participants indicated that they would view CSAM.

Exposure to CSAM often begins during adolescence. In the study by Napier (2022), 9.2% of respondents in the 18–20 age cohort reported first viewing CSAM when they were 14–15 years old, compared to only 2.5% in the 40+ age cohort. A survey conducted by Nurmi and colleagues (2024) among darknet users searching for CSAM revealed that the majority of CSAM users were first exposed to such material during their own childhood. Specifically, 65.3% of respondents first encountered CSAM before turning 18, and 36.7% were 13 years old or younger at the time of their first exposure. A study by Wortley et al. (2024) surveyed 75 male adults who had been arrested, cautioned, or convicted of internet offenses involving indecent images of children or sexual communications with children. The survey found that the youngest age at first contact with CSAM was 10 years, and the mean age was 38 years.

Evidence from large-scale, representative studies confirmed this trend: Studies by Mitchell and colleagues (2007, 2012) in the United States reported an increasing incidence among adolescents of sexual images depicting minors that could be classified as CSAM. Their findings were based on two large, nationally representative telephone surveys of 1500 and 1560 Internet-using youth aged 10–17, respectively. In a U.S. convenience sample of 563 college students, Sabina et al. (2008) found that 15.1% of male and 8.9% of female respondents reported having seen images depicting sexual activity involving children before the age of 18. The study aimed to examine early exposure to online pornography and its potential impact. A meta-analysis found that 12% of adolescents had sent sexually explicit images, videos, and/or messages without the consent of the person in question (Madigan et al., 2018). Among Swedish male high school seniors (*n* = 4339; aged 18) 3.1% had used CSAM, and 17% of frequent pornography users within this study reported CSAM consumption (Svedin et al., 2011). Seto et al. (2015) examined the prevalence, risk factors, and correlates of viewing depictions of adult-child sexual activity using a population-representative sample of 1978 Swedish male third-year high school students (aged 17–20). The study found that 4.2% of participants reported having ever viewed CSAM.

These findings indicate that young people are increasingly exposed to CSAM – not only as potential users of such content, but also as victims of exploitation. For instance, a survey of 9857 students in Switzerland found that approximately 18% of adolescents experienced sexual cybervictimization in 2010 (Averdijk et al., 2011). The normalization and ease of accessing CSAM amplify the problem, as young people may become desensitized to the effects of consuming such material (Prichard et al., 2013; Wortley et al., 2024).

The growing prevalence of CSAM is exacerbated by a lack of understanding of the law. Adolescents in particular are often unaware of the criminal consequences of their actions (Biedermann et al., 2023; Turner et al., 2025). Self-recorded and shared images can be classified as “child pornography,” which can lead to criminal prosecution, e.g., in Australia, the United States and Germany (Biedermann et al., 2023; Moritz & Christensen, 2020; Wolak et al., 2012). The exchange of self-generated sexually explicit content among peers, commonly referred to as “sexting”, is a normative behavior among adolescents and should not be criminalized (Moritz & Christensen, 2020; Quayle, 2022). However, when adolescents consume sexually explicit material depicting children who are significantly younger than they are, this behavior cannot be considered normative. In their typology of offenders, Biedermann et al. (2023) used an age difference of three years to distinguish between peers and victims who are outside the peer age group.

### Risk Factors and Offender Typologies

A recent review by Reichel and colleagues (2024) identified 12 overarching categories of risk factors for online CSAM offending: criminal history, socio-demographics, social relations, sexuality (non-paraphilic), paraphilic interest, CSAM collection, antisociality, childhood experiences, offense-supportive cognitions, health, the Big Five, and other factors that do not fall into any of the above categories. Research, including the study by Wortley et al. (2024), suggests that the use of CSAM is also influenced by situational factors such as stress and low mental well-being.

Among the aforementioned risk factors, paraphilic sexual interest, particularly pedophilia, is one of the most robust predictors of CSAM use (Reichel et al., 2024). However, it is important to note that not all cases of CSAM use can be attributed to pedophilic interests. According to the *Diagnostic and Statistical Manual of Mental Disorders 5*^*th*^ *Ed.* (American Psychiatric Association, 2013), a Pedophilic Disorder is characterized by recurrent, intense sexual fantasies, urges, or behaviors involving sexual activity with a prepubescent child. The diagnosis can only be made if the individual is at least 16 years old and at least 5 years older than the child who is the subject of the fantasies or behaviors. The term hebephilia is used to distinguish it from a sexual interest in pubescent children. Accordingly, relationships between young people within a certain age difference do not reflect a pathological condition or indicate a pedohebephilic preference.

Some of the risk factors identified in adult populations may also be relevant in explaining CSAM use among adolescents. Lehmann and colleagues (2023) discussed various explanatory models, including the motivation-facilitation model (Seto, 2017), which explains CSAM use with factors such as paraphilia, sexual desire, and antisocial tendencies. Klein et al. (2015) suggested that thrill-seeking behavior can play a role in CSAM use, a finding that may be especially relevant during adolescence. Furthermore, Twenge et al. (2017, as cited in Reichel et al., 2024) pointed out that younger age is associated with increased sexual activity.

Research focusing on young people’s engagement with CSAM suggests that their characteristics differ from those of adults. CSAM suspects under 18 years tend to exhibit less entrenched and repetitive offending patterns and are considered less criminally and developmentally motivated than adults (Biedermann et al., 2023). Furthermore, Biedermann and colleagues (2023) typologized underage CSAM suspects as less likely to have had prior run-ins with the law before and note that the victims are typically acquaintances and of the same age. These conclusions are based on German police crime statistics from 2016 to 2021, comparing 1353 suspects in child sexual abuse cases and 8466 suspects in CSAM-related offenses across four federal states. The study contrasted minors (under 18) with adult suspects and examined offender types and offense characteristics, highlighting the need for age-specific prevention and intervention strategies. Stevens et al. (2013) analyzed data from 184 males aged 10–21 years who were referred to a community-based assessment and treatment service for sexually harmful behavior. The study aimed to examine developmental and offense-related characteristics across subgroups, based on victim age and type of offense (contact vs. non-contact), and to explore whether such distinctions are clinically meaningful. A small subgroup (*n* = 6) was identified who had committed exclusively online offenses (“internet offenders”). Compared to others in the sample, they showed higher psychosocial functioning, academic success, and no prior abuse history or delinquent behavior.

### Research Gaps and Objectives of the Study

Most of the findings on personal, social, and psychological factors that foster the use of CSAM are based on studies with adults or do not limit the age of the sample (e.g., Babchishin et al., 2018; Napier et al., 2024). While existing studies provide valuable insights into risk factors and offender profiles, adolescents who engage in CSAM use remain significantly understudied. The above-cited literature on young CSAM users primarily relies on data concerning offending patterns. Specifically, there is a lack of research on minors who seek help voluntarily, before they become known to the law enforcement authorities. The findings presented in the previous sections suggest that there are different types of adolescent CSAM users, who differ, for example, in terms of paraphilic interests, especially pedophilia, as a motive for CSAM use. This study aims to address this research gap by analyzing self-reported data from *n* = 117 adolescents under the age of 18 who voluntarily applied for a therapeutic program aimed at reducing CSAM use. It explores whether distinct subgroups exist within this population, using an exploratory cluster analysis based on psychological characteristics, behavioral indicators, and patterns of CSAM use.

The present study employed multiple measures, derived from known risk factors in adults, to better understand this population and its needs: Mental well-being was included to examine whether psychological distress impacts help-seeking and CSAM use. Relationship status and satisfaction were included as relevant risk factors for CSAM use. Sexual outlet, encompassing masturbation, partnered sexual activity, and pornography use, was examined to gain insight into the participants’ sexuality. Paraphilic interests, including sexual attraction to prepubescent or pubescent body types, were assessed to determine whether the presence of deviant sexual preferences is related to CSAM use. CSAM consumption was evaluated in terms of frequency, duration, and content severity, directly addressing the question of usage patterns. Additional measures capture legal involvement and participants’ behavior toward children to provide critical information for identifying risk-related subgroups.

Given the limited empirical research on help-seeking minors engaged in CSAM use, this study employs an exploratory approach to identify meaningful patterns within this population. As this group has not been extensively studied, no a priori assumptions about the number or composition of potential subgroups were made. Instead, an exploratory cluster analysis was conducted to examine whether distinct profiles emerge based on psychological characteristics, sexual outlet, and CSAM consumption patterns.

Despite the exploratory nature of the study, the following assumptions were made:

• Those seeking help are not primarily individuals who exchange sexually explicit material as peers.
• The majority of help-seekers does not have a criminal record of CSA/M offenses.
• Low mental well-being may be a key motivation for seeking therapeutic help.
• Help-seeking minors do not solely consume “indicative”, “nudist” and “erotic” material (COPINE levels 1–3; Taylor et al., 2001), but rather illegal material that is attributed to higher levels of the COPINE scale.

To the author’s knowledge, this is the first study to explore psychological characteristics in the typology of adolescent CSAM users, aiming to expand the understanding of this group beyond offense-related features. By exploring distinct user profiles, this research seeks to contribute to the development of more targeted support and prevention strategies for minors who engage with CSAM.

## Methodology

This study was based on data collected through the “Troubled Desire” online self-help platform (https://troubled-desire.com). The platform is designed for people who are attracted to children and offers comprehensive information and psychoeducational self-help modules, as well as the opportunity to take a self-test. The self-test is designed to provide users with feedback on their paraphilic interests. During the data collection period, a therapeutic chat was offered, for which interested individuals could sign up via the “Troubled Desire” platform. For this study, the anonymous data from the self-test were combined with the screening questions for participation in the therapeutic chat study.

### Ethics

The intervention study from which the intake assessment the data originate, including the recruitment measures, was approved by the Ethics Committee of Charité – Universitätsmedizin Berlin (06/03/2024, No. EA4/173/23). The analysis of data from participants younger than 18 was not explicitly included in the ethics approval, as minors did not participate in the intervention study. However, all data used in this study were collected with the informed consent of the participants. Data collection was completely anonymous to ensure the privacy and confidentiality of all respondents.

### Study Design

This data were collected as part of a screening process for an online intervention study to evaluate the effectiveness of a therapeutic chat intervention for users of CSAM. Data were collected between October 24, 2023 and September 30, 2024. The data at hand were combined from two distinct yet interconnected components: a publicly accessible self-test and a dedicated application process for participation in the intervention study. This study combined data from these two sources, taking into account only data from individuals under the age of 18.

Participants were recruited through a multi-channel online campaign including: a social media (Facebook, Instagram) campaign featuring short videos and varying text-based messages that either contained facts about CSAM or testimonials, each combined with the reference to the anonymous help offer. Furthermore, posts on forums and blogs affiliated with the self-identified pedophilic community were issued (e.g., Virtuous Pedophiles). The study call was listed on websites that collect resources for individuals with pedohebephilic interests or CSAM users (e.g., prostasia.org). Additionally, keyword-based advertisements were placed on the darknet and on an adult pornography platform, targeting users based on relevant search terms. None of the recruitment measures were specifically targeted at under-18s.

While the intervention study was legally restricted to individuals aged 18 and older, age information was not collected prior to the application. Consequently, minors could technically apply. As a result, they were excluded from the intervention study during the screening process for the therapeutic study but were included in the study at hand.

The self-test and the screening questions for the intervention study were offered in Czech, English, German, Portuguese, and Spanish, corresponding to the languages in which the therapeutic intervention was available. Data collection was completely anonymous. The data from the self-test and the study application were matched using a randomly generated session ID. However, only data from participants who provided formal consent were included in the analysis.

### Sample

**Figure 1.**
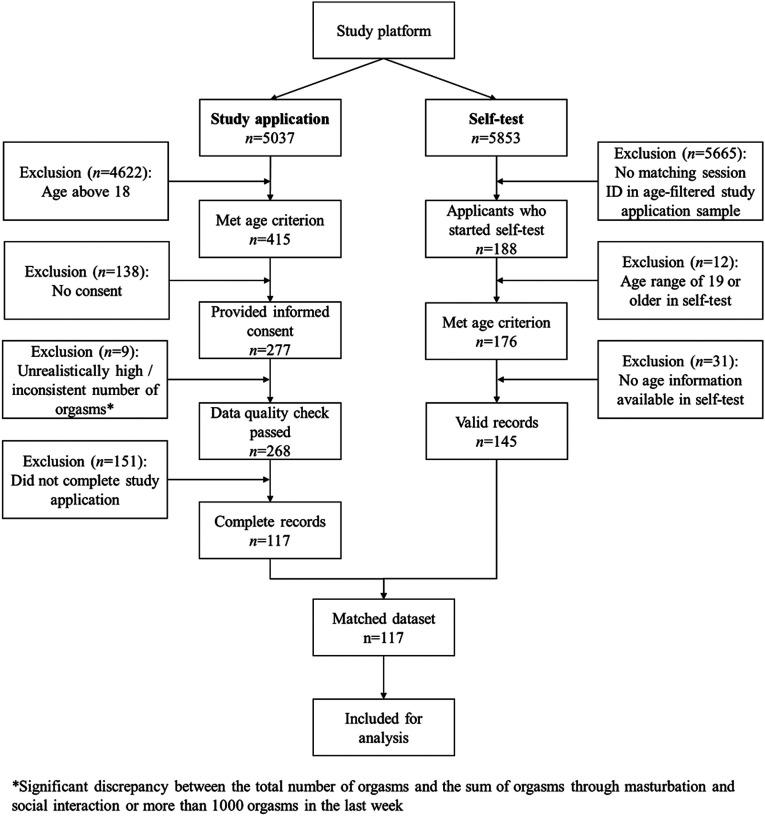
Flowchart of exclusion stages.

Figure 1 illustrates the process that led to the sample for analysis. Of the total *N* = 5037 people who started the screening questionnaire for the therapeutic chat study, *n* = 415 indicated that they were between the age range of 12 and 17 years. Within this group, *n* = 277 individuals gave formal consent to participate in the study. Of these, *n* = 9 records were excluded due to implausible information (a number of more than 1000 orgasms in the last week or significant discrepancy between the total number of orgasms and the sum of orgasms through masturbation and social interaction).

Of those who applied for the intervention study, *n* = 188 people (approximately 70%) completed the self-test. Age matching excluded *n* = 12 individuals who reported an age range of 19 years or older in the self-test and *n* = 31 individuals for whom age information was not available. This left *n* = 145 valid data records after age matching. Finally, all incomplete records from the study application were excluded, resulting in *n* = 117 complete records. Of these *n* = 117 applicants, all started the self-test, *n* = 92 (78.6%) completed the self-test. Table 1 provides an overview of the demographic characteristics of the sample considered.

**Table 1.** Demographic Characteristics of Study Participants.

Variable	Level	*n* (%)
What sex were you assigned at birth, on your original birth certificate?	Female	25 (21.4)
	Male	86 (73.5)
	I do not know	0
	I prefer not to answer	6 (5.1)
What is your gender?	Female	21 (17.9)
	Male	85 (72.6)
	Other	3 (2.6)
	I do not know	2 (1.7)
	I prefer not to answer	6 (5.1)
Level of education	None	32 (27.4)
	School	62 (53)
	Any kind of diploma that proves general qualification for university entrance (e.g., high school diploma)	19 (16.2)
	Vocational education	2 (1.7)
	Bachelor’s or equivalent level	1 (0.9)
	Master’s or equivalent level	1 (0.9)
	Doctoral or equivalent level	0
Stable relationship	No	96 (82)
	Yes	14 (12)
	Unsure	7 (6)
Language	Czech	0
	English	80 (68.4)
	German	20 (17.1)
	Portuguese	5 (4.3)
	Spanish	12 (10.3)

### Measures

#### Mental Well-being

The Warwick-Edinburgh Mental Wellbeing Scale (WEMWBS) by Tennant and colleagues (2007) is an instrument that has been validated in different languages and age groups, including teenagers, to measure mental wellbeing (Clarke et al., 2011; Hunter et al., 2015; Tennant et al., 2007). The scale consists of 14 items with 5 response categories (ranging from *none of the time* to *all of the time*) that are summed into a single score. The items are all positively worded and cover both emotional and functional aspects of mental well-being, e.g., “I’ve been feeling optimistic about the future”, “I’ve been thinking clearly”. Internal consistency of the WEMWBS in the present sample was excellent with Cronbach’s alpha = .91 (95% CI [.89, .93]).

#### Relationship Satisfaction

Relationship satisfaction was assessed with a single item (“How satisfied are you with your relationship or your situation as a single person?”). Responses were given on a 5-point Likert scale, ranging from *very unsatisfied* to *very satisfied*.

#### Orgasms

Open-ended questions about the number of orgasms in the past week were used to record sexual experiences. Participants were asked to report the total number of orgasms achieved (“How many orgasms did you achieve during the last week?”) as well as the number achieved through masturbation (“How many of these were by masturbation?”) and through interaction with other people (“How many of these by sexual interaction with other people?”).

#### Paraphilic Interests

The paraphilic interest survey included questions about sexual preferences related to Tanner stages of both sexes (e.g., “Do you find childlike boys/boys with a prepubescent body type (i.e. no pubic hair, childlike penis or scrotum) sexually arousing?”) and other paraphilias (fetishism, transvestism, masochism, sadism, voyeurism, exhibitionism). If participants reported a paraphilic interest, they were asked if the interest had persisted for at least six months (“Are these fantasies sexually arousing for at least six months?”). For all questions regarding paraphilias, the answer options were *yes* and *no*.

#### Use of Pornographic Materials and CSAM

Participants were asked how often (*never*, *some time ago*, *recently,* or c*urrently*) they used pornographic materials during masturbation and if they had ever used CSAM (“Have you ever in your life looked at images of pre- or early pubertal children for sexual arousal?”, response options: *yes* or *no*). If yes, an adapted version of the COPINE (“Combating Paedophile Information Networks in Europe”, Taylor et al., 2001) scale was used to inquire in detail about the severity of the content consumed. A description of the different COPINE levels is provided in Table 4. In addition, the frequency (“How often have you used these materials during the last two weeks?”, response options: *never*, *once*, *once/twice a week (2/4 times during last two weeks), several times a week (more than four times during last two weeks)* and *everyday*) and the duration of CSAM use (“How much time did you spend in total using these materials during the last two weeks?”) in the last two weeks were assessed.

#### Behavior Toward Children

Participants were asked about various types of contact with children, ranging from everyday encounters to specific sexual acts (see Table 5 for item descriptions). Participants indicated whether such contact had occurred *never*, *some time ago*, *recently,* or *currently*.

#### Legal Status

Participants’ current and past legal status was assessed via self-report using separate items. To capture current legal involvement, participants were asked whether they were currently under investigation, on trial, or serving a sentence (including probation or fines) for child sexual abuse, online or offline, or for using, possessing, or distributing CSAM. Response options for these items were *yes* or *no*. To assess past legal involvement, participants were asked whether they had ever been prosecuted for child sexual abuse or charged with possession, use, or distribution of such material. Response options included *no*, *criminally prosecuted*, and *convicted*.

### Data Analysis

The data analysis began with a descriptive presentation of all the variables collected. Depending on the variable level, the distribution of responses, frequencies, and, where applicable, means and standard deviations were calculated to provide an overview of the distribution of the data.

To identify meaningful subgroups within the help-seeking adolescent sample, an exploratory cluster analysis was conducted. In the fields of criminology and forensic psychology, typological approaches and the search for patterns are frequently employed to better understand heterogeneity within populations, helping to develop targeted prevention and intervention strategies (e.g., Biedermann et al., 2023; Krone, 2004). Cluster analysis is particularly suited for exploratory purposes, as it does not assume that groups are already known but rather serves to discover groups in data (Everitt et al., 2011). The exploratory analysis was conducted using hierarchical clustering with the “hclust” function to identify potential patterns and groupings in the data based on differences. The “complete linkage” method was employed, which considers the maximum distances between groups to form clusters. This method is particularly effective in identifying well-defined, homogeneous groups while mitigating the impact of outliers (Everitt et al., 2011).

The following variables were included in the cluster analysis: mental well-being, relationship satisfaction, frequency of pornography use, frequency and duration of CSAM use in the last two weeks, and self-reported problematic behavior. Binary variables such as paraphilic interests (e.g., sexual attraction to prepubescent or pubescent individuals) and COPINE levels were excluded in the clustering procedure, as the method does not support binary input. Additionally, the participants’ gender was excluded due to unequal distribution and low variance in the sample.

Various statistical tests were used to further analyze and validate the identified clusters: the Chi^2^ test for categorical variables, the Mann-Whitney U test and the Wilcoxon test for ordinal scaled or non-normally distributed metric variables, and the Fisher test for small samples or rare categories. Variables that were not included in the cluster analysis were subsequently tested for significant differences between clusters to explore whether certain characteristics (e.g., paraphilic interests, legal involvement) varied meaningfully across the identified subgroups. To reduce the risk of Type I errors due to multiple comparisons, the Bonferroni correction was applied as needed. All statistical analyses (including graphs) were performed using the “R Statistics” software (R Core Team, 2023). In addition, “ChatGPT 4o” (OpenAI, 2024) was used to assist in code generation for the analyses. The author takes responsibility for the integrity of the data, the accuracy of the data analyses, and has made every effort to avoid inflating statistically significant results.

## Results

### Mental Well-being

On average, the total score of *M* = 39.93 (*SD* = 10.96) on the WEMWBS indicates a below average level of well-being compared to student populations where the mean total score was between 48.8 and 50.7 (Clarke et al., 2011; Taggart et al., 2008). In other populations, WEMWBS scores tend to be lower, as observed in French adolescents with psychiatric disorders during the COVID-19 lockdown, where 16 to 17-year-olds had a mean score of *M* = 39.31 (*SD* = 11.94) and 18 to 19-year-olds scored *M* = 41.06 (*SD* = 10.62), as well as in adolescents at risk of experiencing mental health difficulties who reported an average WEMWBS score of *M* = 45.99 (*SD* = 10.38) (Kuosmanen et al., 2017; Orfeuvre et al., 2022).

### Relationship Satisfaction

Relationship satisfaction was analyzed according to participants’ relationship status. The majority of participants who were not in a stable relationship (*n* = 96; 82%) reported moderate relationship satisfaction (*M* = 2.74, *SD* = 0.99). In contrast, the participants in stable relationships (*n* = 14; 12%) reported an average relationship satisfaction rating of *M* = 3.57 (*SD* = 1.34). Participants who were uncertain about their relationship status (*n* = 7; 6%) reported the lowest relationship satisfaction (*M* = 2.57, *SD* = 0.98). A Kruskal-Wallis test revealed a significant difference in relationship satisfaction between participants with different relationship statuses, χ^2^(2) = 17.34, *p* < .001. Post-hoc Dunn tests with Bonferroni correction revealed that participants in stable relationships reported significantly higher relationship satisfaction compared to those not in a relationship (*Z* = 4.16, *p* < .001). However, no significant differences were found between participants in a stable relationship and those uncertain about their relationship status (*Z* = 2.32, *p* = .061) or between participants without a stable relationship and those uncertain (*Z* = -0.18, *p* = 1.000).

### Orgasms

The total number of orgasms in the last week was on average *M* = 6.34 (*SD* = 8.15), ranging from *min* = 0 to *max* = 50. Differentiated by type of experience, the participants reported an average of *M* = 6.03 orgasms (*SD* = 7.98; *min* = 0; *max* = 50) through masturbation. The mean number of orgasms from sociosexual interactions was *M* = 0.54 (*SD* = 2.13; *min* = 0; *max* = 15).

### Paraphilic Interests

The preference for specific developmental stages, differentiated by gender of body scheme, is shown in Table 2. Overall, 46.2% (*n* = 54) of the participants indicated a preference for females in the infantile body schema, while 29.1% (*n* = 34) indicated this preference for males. Preferences lasting longer than six months were also more common for female (*n* = 49; 41.8%) than male (*n* = 29; 24.8%) Tanner stage 1 body schemas. Preferences for pubescent (Tanner stages 2–3) and adult (Tanner stages 4–5) body schemas were also reported, although the frequency differed by gender of body scheme and developmental stage (see Table 2). Other paraphilic preferences were also reported (see Table 3). The most commonly reported preferences were voyeurism (*n* = 49; 41.9%), sadism and masochism (*n* = 47; 40.2%).

**Table 2.** Preference by Gender of Body Scheme and Stage of Development.

Tanner stadiums	Gender	Preference present	Preference 6+ months
1 (prepubescent)	Female	54 (46.2%)	49 (41.8%)
	Male	34 (29.1%)	29 (24.8%)
2–3 (pubescent)	Female	56 (47.9%)	52 (44.4%)
	Male	24 (20.5%)	23 (19.7%)
4–5 (mature)	Female	64 (54.7%)	58 (49.6%)
	Male	30 (25.6%)	26 (22.2%)

**Table 3.** Paraphilic Interests.

Paraphilia	Preference present	Preference 6+ months
Exhibitionism	19 (16.2%)	19 (16.2%)
Fetishism	19 (16.2%)	13 (11.1%)
Masochism	47 (40.2%)	39 (33.3%)
Sadism	47 (40.2%)	43 (36.8%)
Transvestite fetishism	27 (23.1%)	24 (20.5%)
Voyeurism	49 (41.9%)	43 (36.8%)

### Use of Pornographic Materials and CSAM

34% (*n* = 4) of participants reported never using pornography, 10.3% (*n* = 12) had used pornography some time ago, 16.2% (*n* = 19) recently, and 50.4% (*n* = 59) reported currently using pornographic materials. *n* = 23 participants did not answer this question.

**Figure 2.**
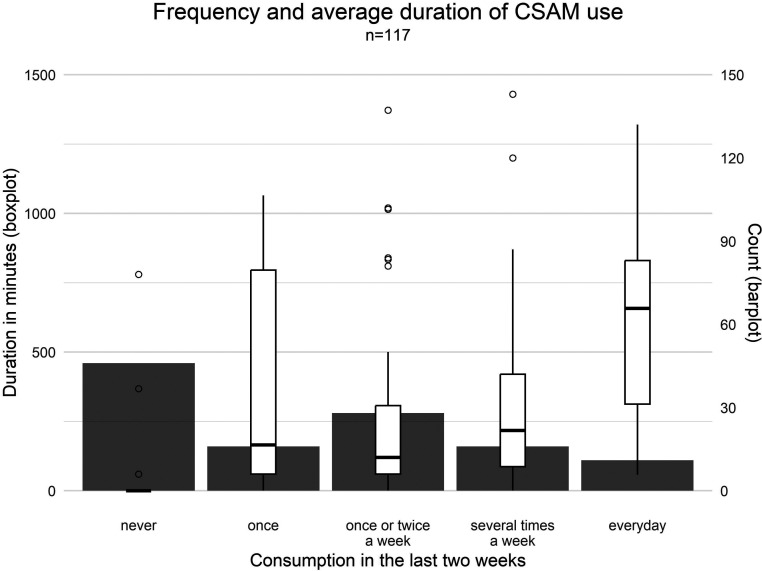
Frequency and duration of CSAM use in the past two weeks.

77.8% (*n* = 91) of participants reported ever using CSAM. The duration and frequency of use varied, with an overall mean duration of use of *M* = 257.41 minutes (*SD* = 373.7; *min* = 0, *max* = 1430). The specific distributions of duration by frequency of use in the last two weeks are shown in Figure 2. Severity of use was assessed using the COPINE scale. The frequencies of responses in the different categories refer to the total sample (*n* = 117) and are shown in Table 4. The most prevalent levels of use were content focusing on the genitals, buttocks, or breasts (level 6, *n* = 52; 44%), content featuring sexual acts between children and adults (level 9, *n* = 48; 41%) and sexual touching of or by adults (level 8, *n* = 47; 40.2%).

**Table 4.** Severity of the Materials Consumed (Adapted COPINE Scale).

COPINE level		Consumption reported
1	Clothed children in everyday situations	29 (24.8%)
2	Unclothed/lightly clothed children in everyday situations	45 (38.5%)
3	Secretly taken pictures of unclothed/lightly clothed children in everyday situations	34 (29.1%)
4	Non-sexualized posing	35 (29.9%)
5	Sexualized posing	41 (35%)
6	Focus on genitals, buttocks or breasts	52 (44%)
7	Sexual activity alone or with other children	45 (38.5%)
8	Sexual touching of/by adults	47 (40.2%)
9	Sexual act with adults	48 (41%)
10	Children suffer pain	25 (21.4%)
11	Sexual acts of children with animals	15 (12.8%)

### Behavior Toward Children

The descriptive analysis of behavior toward children indicated that the most common interactions are everyday encounters and looking at children without any attempt at sexual activity. Overall, sexual behavior toward children is rarely reported. Table 5 provides an overview of all the reported behaviors.

**Table 5.** Behavior Toward Children.

Item	Never	Some time ago	Recently	Currently
I encounter children in everyday life (…)	18 (15.4%)	9 (7.7%)	23 (19.7%)	43 (36.8%)
I have looked at children without any attempt at sexual activity (…)	15 (12.8%)	12 (10.3%)	30 (25.6%)	36 (30.8%)
I had physical contact with children to arouse myself sexually (…)	67 (57.3%)	12 (10.3%)	4 (3.4%)	9 (7.7%)
I watched children in intimate situations to get sexually aroused	53 (45.3%)	15 (12.8%)	14 (12%)	10 (8.5%)
I have taken photos or videos of children in intimate situations to arouse myself sexually (visible genitals/anal area)	75 (64.1%)	5 (4.3%)	7 (6%)	5 (4.3%)
I have performed sexual acts without physical contact with a child (…)	70 (59.8%)	11 (9.4%)	3 (2.6%)	8 (6.8%)
I have caused a child to perform sexual acts on themselves or another child while I was watching	80 (68.4%)	7 (6%)	1 (0.9%)	4 (3.4%)
I have shown sexual behavior in the presence of children (…)	75 (64.1%)	8 (6.8%)	3 (2.6%)	6 (5.1%)
To get sexually aroused, I touched children’s genitals/bottom	73 (62.4%)	9 (7.7%)	4 (3.4%)	6 (5.1%)
I have orally stimulated the genitals/buttocks of a child (…)	81 (69.2%)	5 (4.3%)	1 (0.9%)	5 (4.3%)
I Made a child perform sexual acts on me	80 (68.4%)	6 (5.1%)	2 (1.7%)	4 (3.4%)
I have inserted my penis into a child’s mouth/I have made a child kiss or lick my vagina	83 (70.9%)	3 (2.6%)	3 (2.6%)	3 (2.6%)
I have inserted my finger, penis or an object into a child’s bottom or vagina	82 (70.1%)	5 (4.3%)	2 (1.7%)	3 (2.6%)

### Legal Status

The majority of participants (*n* = 89; 76.1%) reported that they had never been prosecuted for child sexual abuse. Two individuals (1.7%) reported that they had been convicted in the past, and one participant (0.9%) reported that they had been prosecuted. Current criminal investigations or proceedings for child sexual abuse, either online or offline, were reported by *n* = 4 individuals (3.4%). Investigations, proceedings, or penalties related to the use, possession, or distribution of CSAM were reported by *n* = 7 respondents (6%).

### Cluster Analysis

**Figure 3.**
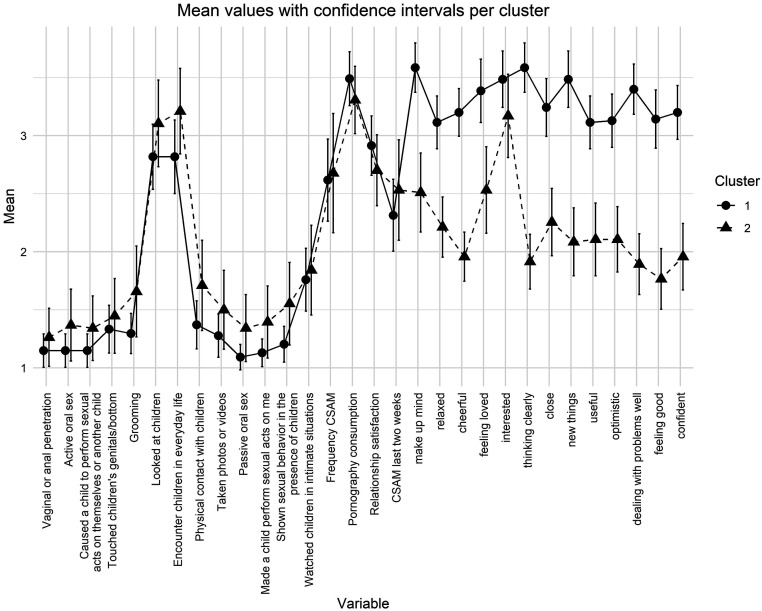
Means of identified clusters.

An exploratory hierarchical cluster analysis was conducted to identify and describe distinct groups within the sample. Two clusters emerged from the exploratory analysis. The means of the variables in each cluster, along with their confidence intervals, are presented in Figure 3. A closer look at the clusters shows that psychological well-being is significantly higher in Cluster 1 than in Cluster 2.13 of the 14 items of the WEMWBS showed significantly higher scores in favor of Cluster 1 (see the attached Supplemental Material for details).

There were trends indicating differences in encounters with children in everyday life (*U* = 832, *p* = .076) and passive oral sex (*U* = 918, *p* = .098), although the clusters did not differ significantly on these variables. These results suggest possible differences between the clusters, but do not show strong statistical evidence. In addition, the sum of problematic behaviors per participant was compared between the two clusters. The analysis revealed no statistically significant difference between the clusters with regard to behaviors toward children (*U* = 886.5, *p* = .213). No differences between the clusters were found except for the self-identified gender. The Chi-square test showed a significant difference in self-identified gender distribution between the clusters (*χ*^2^ = 4.73, *p* = .031), a finding that was subsequently validated through the implementation of the Fisher test (*p* = .015). This indicates that Cluster 2 exhibits a greater degree of diversity in terms of self-identified gender distribution. There was no significant difference between Cluster 1 and Cluster 2 in terms of the maximum COPINE level reported (*W* = 1848.5, *p = .*33). This suggests that the severity of CSAM consumed, as measured by the highest reported COPINE level, did not differ substantially between the identified subgroups.

## Discussion

The results of the study reveal the characteristics of underage help-seekers who have engaged with CSAM and have voluntarily sought support. The findings are discussed below, structured along CSAM usage patterns and the key categories of risk factors:

### CSAM Use

Nearly 80% of respondents stated having ever used CSAM. Approximately 30–40% of the respondents reported using material ranging from COPINE levels 2 to 9, with significant variation in frequency and duration of use. A total of 13.7% (*n* = 16) reported using CSAM once in the past two weeks, while 23.9% (*n* = 28) reported using CSAM two to four times within the same period. 13.7% (*n* = 16) reported consuming CSAM more than four times in the past two weeks, and 9.4% (*n* = 11) reported daily use. The duration of CSAM use also showed considerable variability, with an average duration of *M* = 257.41 minutes (*SD* = 373.7) over the past two weeks. Despite this variability, the data indicates a pattern of regular use, with less than half of the respondents (39.3%; *n* = 46) reporting no use of CSAM in the past two weeks. The most frequently used content fell under level 6 (“Focus on genitals, buttocks, or breasts”; reported by 44%), level 9 (“Sexual acts between children and adults”; reported by 41%), and level 8 (“Sexual touching involving adults”; reported by 40.2%). The least used content included level 10 (“Children experiencing pain”) and level 11 (“Sexual acts involving children and animals”). It can therefore be assumed that the respondents do not only use material that has been exchanged between peers in the context of sexting.

### Criminal History

In terms of CSAM-related offending, 94% of the participants belonged to the Dunkelfeld category, meaning they had no official criminal record. This aligns with findings from Biedermann et al. (2023), who also reported that minors suspected of CSAM offenses rarely exhibited multiple prior offenses. Hands-on or contact offenses involving children, which are characterized by physical contact with the victim (Seto & Eke, 2015), were reported by only a small number of participants, while the majority admitted to using CSAM. These findings are consistent with previous research suggesting that hands-on or mixed offenders are more likely to have prior sexual offenses compared to CSAM-only offenders (Babchishin et al., 2015).

### Social Relations

The study incorporated relationship status and satisfaction as social indicators. The majority of the participants were not in stable relationships, which is not surprising given their young age. However, those in stable relationships reported significantly higher satisfaction with their relationship status compared to those who were not. Despite this difference, relationship satisfaction did not vary significantly between the two clusters. Consequently, no definitive conclusions can be drawn regarding the protective effect of stable relationships in this particular sample.

### Sexuality

The majority of the study’s participants identified as male and gynephilic. The number of orgasms achieved through masturbation in the past week ranged widely (from 0 to 50) which points to substantial individual differences in sexual preoccupation and sex drive. Orgasms resulting from sociosexual interactions were rarely reported. Approximately half of the participants reported currently using pornographic materials, suggesting that solitary sexual activity and the use of visual stimuli play a significant role in their sexual behavior.

### Paraphilic Interests

The study confirms that paraphilic sexual interests play a role in CSAM use among minors. While nearly half of the sample reported teleio-gynephilic preferences, many also reported pedophilic or hebephilic interests. However, it is crucial to note that a hebephilic interest in adolescents is not a paraphilia, but rather an age-appropriate or developmentally appropriate interest. This data cannot be investigated further, because no specific age information about the participants is available, making it impossible to differentiate between 12 and 17-year-olds, for instance. Additionally, approximately a third of respondents reported other paraphilic interests, such as voyeurism, sadism, and masochism.

### Health

Mental well-being, measured with the WEMWBS, varied substantially. On average, participants scored below the population norm for adolescents and students. However, a total of *n* = 70 participants (59.8%) were assigned to Cluster 1. This group demonstrated higher levels of mental well-being (WEMWBS_total_ *M* = 46.24, *SD* = 7.64). Conversely, *n* = 47 participants (40.2%) were allocated to Cluster 2. These individuals reported a low psychological well-being (WEMWBS_total_ *M* = 30.53, *SD* = 8.05). Thus, a possible relation between mental well-being and help-seeking behavior as well as CSAM use cannot be confirmed.

### Two Distinct Subgroups

The exploratory cluster analysis revealed two distinct subgroups within the sample of help-seeking adolescents who engage with CSAM. Cluster 1, comprising approximately 60% of the sample, was characterized by higher mental well-being and fewer self-reported problematic behaviors toward children. In contrast, Cluster 2 (approximately 40%) showed significantly lower mental well-being and a slightly elevated – though not statistically significant – tendency toward problematic behaviors involving children. Cluster 2 also exhibited greater diversity in terms of gender identity.

### Implications for Interventions

In general, consistent with the findings of Insoll et al. (2022), the voluntary engagement with support services suggest that the respondents are aware of their problematic CSAM use and demonstrate a willingness to change. The number of applications from minors willing to participate (*n* = 277 in approximately 12 months) highlights that there is a need for support services for young people using CSAM. In the study by Wortley et al. (2024), access to therapeutic support was the most frequently mentioned factor that could potentially prevent participants from using CSAM.

Furthermore, the results of this study provide a basis for the development of target-group-specific intervention measures. It is evident that the offer was particularly attractive to young people because the intervention was available online and anonymously, which significantly lowers the barriers to seeking help. For the target group of minors, anonymity may be even more important than for adults; stigmatization and labeling, which are known to be potentially self-fulfilling prophecies with lasting negative effects on the lives of those affected, can be avoided. Additionally, today’s generation of digital natives is highly comfortable with the digital realm, making online solutions for sensitive topics a preferred choice. In contrast, offline services would likely require the involvement or knowledge of parents or other caregivers, which could act as a barrier, deterring many young people from seeking support. The help-seeking behavior demonstrated by voluntarily completing the questionnaire should be actively rewarded in order to avoid frustration and encourage participation. Positive reinforcement, such as access to further support or appreciation for participation, could contribute to motivate individuals to seek help at an early stage.

As in many studies, such as Napier et al. (2024), the majority of the sample consisted of male CSAM users. However, the present study showed that approximately 20% of respondents identified as female or non-binary. These observations may also be artifacts of the survey methodology. Nevertheless, future studies and prevention efforts should be designed to be more inclusive.

About half of the participants reported having a pedohebephile sexual preference. Looking at the COPINE levels of the material used, as well as the duration and frequency of CSAM use, it becomes clear that in many cases these are not exclusively “youth typical” incidents such as non-consensual image sharing. Accordingly, it must be assumed that the target group reached consists primarily of individuals who genuinely have a problem that necessitates treatment and cannot be addressed merely by informing them of the legal consequences of their actions. Consequently, treatment programs for adolescents must go far beyond educational aspects and be flexible enough to respond to individual needs.

Taking the clusters into account, it would be advisable to offer an intervention that focuses on behavior regulation, psychoeducation, and sexual impulse management for individuals with an average level of mental well-being (Cluster 1). Individuals with low mental well-being (Cluster 2) would likely benefit more from an intervention that focuses on improving well-being to build resources for changing problematic behaviors.

### Limitations of the Study

The present study provides insight into the behaviors and characteristics of underage CSAM users, a group that has been underrepresented in research to date. Nevertheless, there are a number of limitations that restrict the generalizability and validity of the findings. First, the participants in this study are individuals who voluntarily sought help because they are aware of the problematic nature of their behavior and strive to change. Thus, there is a self-selection bias that limits the validity of the study results to CSAM users who seek help. No statements can be made about those who have no intention of seeking help or who were not reached by the recruitment efforts, although this is presumably a much larger sample.

The study is based on self-reported data, which may be biased by social desirability, shame, or ambiguities in self-assessment. Objective validation is not possible. Particularly in the sensitive area of CSAM use, participants may have provided incomplete or inaccurate information for fear of stigmatization or legal consequences. In addition, to protect the anonymity of help-seekers, there is no control mechanism to prevent the same person from completing the survey more than once.

In addition, the questionnaire was originally designed for adults, and some relevant constructs were not adequately assessed for a younger population. For example, it is unknown whether participants themselves had been affected by sexual cybervictimization. This limits the ability to examine the dual role of victim and perpetrator, which is particularly relevant in the context of adolescent CSAM use.

Another limitation concerns the measurement of age. Instead of collecting exact age data, the questionnaire offered only age ranges as response options. This compromises the capacity to accurately depict the age distribution within the sample and may have influenced the outcomes of the cluster analysis. Furthermore, it limits the interpretability of participants’ sexual preferences, especially with regard to hebephilia.

## Conclusion

This study underscores the importance of a differentiated and person-centered understanding of CSAM, particularly in the context of adolescents. Distinguishing between typical adolescent behavior and more persistent or deviant patterns is essential to inform prevention strategies that are developmentally appropriate and targeted. This includes differentiating between peer-related sexting behavior and consumption of exploitative material involving much younger children.

As Wolak and colleagues (2012) point out, effective responses require differentiated approaches to law enforcement, prevention, and education. Turner et al. (2025) emphasize the substantial involvement of under 18s in CSAM cases, with about 50% of perpetrators known to the victim being under 18. Similarly, Biedermann et al. (2023) found that in 30% of the cases considered suspects were under 18 years old. These findings highlight a significant window for primary and secondary prevention that intervenes before the justice system becomes involved.

Despite this urgency, support options for minors are limited (Gannoni et al., 2023). The present study demonstrates that young people are indeed willing to seek help. However, this motivation is often not met with accessible, age-appropriate services. As Insoll et al. (2022) report, nearly half (47.7%) of respondents in their study experienced barriers when seeking help, most commonly fear of legal consequences (79.6%), fear of stigmatization (75.7%), and fear of being rejected by professionals (50.5%). These barriers are exacerbated by a lack of anonymous, low-threshold support services specifically designed for minors.

Finally, as Nurmi et al. (2024) argue, tackling the global rise in CSAM use must be recognized as a public health challenge. Therapeutic support was identified by CSAM users themselves as the most promising deterrent to continued use (Wortley et al., 2024). Therefore, it is essential that prevention and intervention efforts adopt a comprehensive approach.

## Supplemental Material

Supplemental Material - Adolescents in the Dunkelfeld: A Study of Help Seeking Minors Who Use Child Sexual Abuse Materials Supplemental Material for Adolescents in the Dunkelfeld: A Study of Help Seeking Minors Who Use Child Sexual Abuse Materials by Viola Westfal in Sexual Abuse
