# Supplementary material for: Adolescents in the Dunkelfeld: A Study of Help Seeking Minors Who Use Child Sexual Abuse Materials
Source: Sex Abuse. 2025 Jun 16;38(2):187–211. doi: 10.1177/10790632251351254 (PMC12804393; doi:10.1177/10790632251351254)
Supplement: Supplemental Material - Adolescents in the Dunkelfeld: A Study of Help Seeking Minors Who Use Child Sexual Abuse Materials [file sj-pdf-1-sax-10.1177_10790632251351254.pdf]

**Variable**	***U***	***p*-value**
Relationship satisfaction	1828.5	0.282
CSAM last two weeks	1528	0.499
WEMWBS optimistic	2532.5	0.0*
WEMWBS useful	2545	0.0*
WEMWBS relaxed	2494.5	0.0*
WEMWBS interested	1864	0.206
WEMWBS energy	2543.5	0.0*
WEMWBS dealing with problems well	2853.5	0.0*
WEMWBS thinking clearly	2964.5	0.0*
WEMWBS feeling good	2778.5	0.0*
WEMWBS close	2438	0.0*
WEMWBS confident	2672.5	0.0*
WEMWBS make up mind	2504	0.0*
WEMWBS feeling loved	2248	0.001*
WEMWBS new things	2707	0.0*
WEMWBS cheerful	2806	0.0*
Pornography consumption	1200.5	0.256
Frequency CSAM	721	0.941
Encounter children in everyday life	832	0.076
Looked at children	847	0.104
Physical contact with children	917.5	0.273
Watched children in intimate situations	1025.5	1
Taken photos or videos	963	0.464
Grooming	944.5	0.39
Caused a child to perform sexual acts on themselves or another child	930	0.195
Shown sexual behavior in the presence of children	914.5	0.193
Touched children's genitals/bottom	1006.5	0.831
Active oral sex	953	0.307
Made a child perform sexual acts on me	925	0.173
Passive oral sex	918	0.098
Vaginal or anal penetration	983	0.533
WEMWBS total	3062.5	0.0*
Problematic behaviors total	886.5	0.213

* Statistically significant difference ($p < .001$) between cluster 1 and 2
